# Periodontal ligament stem cell-derived exosome-loaded Emodin mediated antimicrobial photodynamic therapy against cariogenic bacteria

**DOI:** 10.1186/s12903-024-04062-7

**Published:** 2024-03-07

**Authors:** Maryam Pourhajibagher, Abbas Bahador

**Affiliations:** 1 Dental Research Center, Dentistry Research Institute, Tehran University of Medical Sciences, Tehran, Iran; 2 Department of Microbiology, School of Medicine, Tehran University of Medical Sciences, Tehran, Iran; 3 Fellowship in Clinical Laboratory Sciences, BioHealth Lab, Tehran, Iran

**Keywords:** Exosome, Emodin, Antimicrobial photodynamic therapy, Bioinformatics tools

## Abstract

**Background:**

This study was conducted to investigate the efficiency of periodontal ligament (PDL) stem cell-derived exosome-loaded Emodin (Emo@PDL-Exo) in antimicrobial photodynamic therapy (aPDT) on *Streptococcus mutans* and *Lactobacillus acidophilus* as the cariogenic bacteria.

**Materials and methods:**

After isolating and characterizing PDL-Exo, the study proceeded to prepare and verify the presence of Emo@PDL-Exo. The antimicrobial effect, anti-biofilm activity, and anti-metabolic potency of Emo, PDL-Exo, and Emo@PDL-Exo were then evaluated with and without irradiation of blue laser at a wavelength of 405 ± 10 nm with an output intensity of 150 mW/cm^2^ for a duration of 60 s. In addition, the study assessed the binding affinity of Emodin with GtfB and SlpA proteins using in silico molecular docking. Eventually, the study examined the generation of endogenous reactive oxygen species (ROS) and changes in the gene expression levels of *gelE* and *sprE*.

**Results:**

The study found that using Emo@PDL-Exo-mediated aPDT resulted in a significant decrease in *L. acidophilus* and *S. mutans* by 4.90 ± 0.36 and 5.07 log_10_ CFU/mL, respectively (*P* < 0.05). The study found that using Emo@PDL-Exo for aPDT significantly reduced *L. acidophilus* and *S. mutans* biofilms by 44.7% and 50.4%, respectively, compared to untreated biofilms in the control group (*P* < 0.05). Additionally, the metabolic activity of *L. acidophilus* and *S. mutans* decreased by 58.3% and 71.2%, respectively (*P* < 0.05). The molecular docking analysis showed strong binding affinities of Emodin with SlpA and GtfB proteins, with docking scores of -7.4 and -8.2 kcal/mol, respectively. The study also found that the aPDT using Emo@PDL-Exo group resulted in the most significant reduction in gene expression of *slpA* and *gtfB*, with a decrease of 4.2- and 5.6-folds, respectively, compared to the control group (*P* < 0.05), likely due to the increased generation of endogenous ROS.

**Discussion:**

The study showed that aPDT using Emo@PDL-Exo can effectively reduce the cell viability, biofilm activity, and metabolic potency of *S. mutans* and *L. acidophilus*. aPDT also significantly reduced the expression levels of *gtfB* and *slpA* mRNA due to the increased endogenous ROS generation. The findings suggest that Emo@PDL-Exo-mediated aPDT could be a promising antimicrobial approach against cariogenic microorganisms.

## Introduction

Dental caries, commonly known as tooth decay, is a multifactorial disease that results from the interaction between various environmental, behavioral, and biological factors [1]. One of the most important biological factors contributing to dental caries is the presence of cariogenic bacteria in the oral cavity [2]. Dental caries is caused by a mixture of microorganisms. Specific types of acid-producing bacteria, especially *Streptococcus mutans*, as early colonizers and *Lactobacillus acidophilus* as the second major contributor to dental caries play a significant role in the initiation and progression of dental caries, respectively [3].

*S. mutans* has a central role in the etiology of dental caries. It able to form extracellular polysaccharides (EPS) such as glucans and fructans in the presence of sucrose, fructose and glucose. Glucans and fructans are homopolysaccharides of glucose and fructose, respectively. Glucans and fructans are produced by glucosyltransferases (Gtf) and fructosyltransferases (Ftf), respectively which enable the bacterial cells to adhere to the enamel salivary pellicle and form biofilms known as dental plaque which contribute to the initiation of caries. In the formation of microbial biofilms and dental plaque, *S. mutans* is a primary colonizer, sometimes referred to as "pioneer species [3]. *S. mutans* produces three types of Gtf enzymes including GtfB, GtfC, and GtfD. GtfB is the most abundant and active Gtf responsible for the synthesis of water-insoluble glucans. GtfB plays a key role in bacterial colonization on tooth surfaces. On the other hand, its expression increases with the growth of *S. mutans* in a biofilm structure. Therefore, GtfB plays an important role in the pathogenesis of dental caries by contributing to the pathogenicity of *S. mutans* and the formation of cariogenic biofilms [4–7]. *S. mutans* is highly proficient at producing acid from dietary sugars, leading to the creation of an acidic environment within dental plaque. This acidic environment poses a risk for the demineralization of enamel and the development of cavities. The bacterium's capacity to survive and flourish in acidic pH and its efficient sugar uptake further emphasize its significance in the development of dental caries [3].

*Lactobacillus* spp. especially *L. acidophilus* is considered the second major contributor to dental caries, particularly in the development of advanced caries lesions. These species have been found in carious lesions and dental plaque, and their acidogenic properties have been linked to an increased risk of caries. Unlike *S. mutans*, which is a cariogenic partner, *L. acidophilus* does not adhere efficiently to the tooth surface alone. However, in the presence of *S. mutans* and other primary colonizers, *L. acidophilus*'s ability to establish on the tooth surface can be significantly enhanced. Mechanical retention, such as trapping in food remnants and plaque biofilms, and especially the glucan scaffold of *S. mutans*, is a significant factor contributing to *L. acidophilus* establishment. Additionally, adhesin-receptor-mediated active intercellular contact and probably metabolites-associated interactions are involved in the *S. mutans*-facilitated *L. acidophilus* adherence and persistence [8]. The surface layer proteins (Slps) are a major component of the cell surface of *L. acidophilus* which involved in a variety of biological functions, including adhesion to host tissues, mediating cell–cell interactions, biofilm formation, modulation of the immune system, and bacterial protection against environmental stressors. The role of Slps in the attachment of *L. acidophilus* on the tooth surface is significant, especially in the presence of other primary colonizers such as *S. mutans* in the context of dental caries. Furthermore, the presence of proteinaceous fibers in *L. acidophilus* isolated from patients with severe early-childhood caries, as well as their ability to adhere to surfaces coated with type I collagens, underscores the importance of Slps in the establishment and persistence of *L. acidophilus* in the complex microbiota of dental plaque on the tooth surface [9–14].

Antimicrobial photodynamic therapy (aPDT) is a promising treatment modality that uses a light source at a specific wavelength in combination with a photosensitizer, which generates reactive oxygen species (ROS) that can kill microorganisms [15]. The effectiveness of aPDT depends on several factors, including the concentration of the photosensitizer, the wavelength and intensity of the light, and the duration of exposure. Additionally, the specific microbial species and their susceptibility to ROS can also affect the efficacy of aPDT [16].

Emodin, with the chemical name 3-methyl-1,6,8-trihydroxyanthraquinone, is a well-known natural compound of the anthraquinone group that is approved by the Food and Drug Administration (FDA) [17]. It is extracted from plants such as *Rheum palmatum* and *Polygonum cuspidatum*. Pharmacological information shows that Emodin has a wide range of biological activities, including antibacterial, antiviral, and anticancer effects, and its effect on anti-biofilm microbial activity has also been reported. Moreover, Emodin has been found to be non-toxic to mammalian cells at therapeutic concentrations, which is an important consideration for clinical [18–20]. To overcome these adverse effects, scientists have suggested the use of biological nano-biocarriers. Exosomes, which are small extracellular vesicles that range in size from 30–100 nm and contain various bioactive molecules including proteins, lipids, and nucleic acids, have been proposed as a promising solution due to their ability to penetrate biological barriers and target specific cells and tissues [21, 22]. These nano-biocarriers can carry biologically active substances within their internal space or on their surface molecules, and their hydrophilic and hydrophobic characteristics make them capable of transporting various molecules [23]. The unique features of Exosomes make them an ideal vehicle for drug delivery, offering benefits such as low immunogenicity, long-term safety, absence of toxicity, high stability in physiological fluids, and the ability to carry specific molecules [22–24]. Exosomes derived from different sources such as periodontal ligament (PDL) stem cells have shown great potential in treating various diseases. In fact, PDL stem cell-derived exosomes (PDL-Exos) have been investigated as a source of drug delivery, with the potential to improve drug bioavailability and efficacy, reduce toxicity, and improve pharmacokinetic profiles [25, 26]. No studies have been conducted to investigate the antimicrobial properties of aPDT utilizing Emo@PDL-Exo according to existing literature. Therefore, the aim of this study is to examine the potential antimicrobial effect and anti-metabolic properties of Emo@PDL-Exo-mediated aPDT on *S. mutans* and *L. acidophilus*. The study also measures the endogenous ROS production and evaluates the ability of Emodin to bind to GtfB and SlpA proteins using molecular docking method. Additionally, the research determines any changes in the gene expression levels of *gtfB* and *splA* in the treated *S. mutans* and *L. acidophilus*, respectively. The null hypothesis suggests that the use of Emo@PDL-Exo-mediated aPDT will result in a significant decrease in cell viability, metabolic activity, and a downregulation of virulence factors in both *S. mutans* and *L. acidophilus* following the generation of endogenous ROS.

## Materials and methods

### Isolation of PDL stem cells

To obtain PDL stem cells, healthy third molars were extracted for orthodontic reasons from patients who provided informed consent at Tehran University Dental Clinic, Tehran, Iran. The freshly extracted teeth were transported to a research lab in Hank's balanced salt solution containing penicillin/streptomycin. Two-thirds of the root surface were scraped to remove tissue, which was then washed in Dulbecco's Modified Eagle's Medium (DMEM; Gibco Inc. USA). The tissue was cut into small pieces and digested in an enzyme solution of collagenase I and dispase in DMEM for 20 min at 37 °C. After centrifugation, the pellet was transferred to 35 mm culture dishes containing DMEM medium supplemented with fetal bovine serum and penicillin/streptomycin and incubated at 37 °C with 5% CO_2_ and 95% humidity. The cells were subcultured in fresh medium after 7 days, and the cell culture supernatant of cells at the 2nd and 3rd passage was used to isolate Exos.

### Exosome extraction and characterization

The extraction of Exos from PDL stem cells was carried out using the EXOCIB kit (Cib Biotech, Iran) according to the manufacturer's instructions. In brief, the PDL stem cells were subjected to centrifugation at 3000 rpm for 20 min to separate particles and debris. Subsequently, the PDL stem cells were mixed with reagent A (the exosome precipitation solution) and incubated overnight at 4 °C, followed by centrifugation at 3000 rpm for 40 min to remove the supernatant. The resulting PDL-Exo plate was suspended in phosphate-buffered saline (PBS; pH 7.4) and stored at -70 °C. The protein content of the PDL-Exos was measured using a bicinchoninic acid (BCA) protein assay kit (DNAbiotech Co., Tehran, Iran).

The extracted PDL-Exos were labeled with anti-CD81 and analyzed using a FACSCalibur flow cytometer. The morphological characteristic of the PDL-Exos was evaluated by transmission electron microscopy (TEM; Zeiss EM10C, Germany) with an accelerating voltage of 100 kV. In addition, size distribution of isolated exosomes was measured using dynamic light scattering (DLS; Zetasizer Nano-ZS system, Malvern Instruments, UK).

### Preparation of Emo@PDL-Exo

To prepare Emo@PDL-Exo, Emodin with a purity of 99% (Sigma, Germany) was mixed with extracted PDL-Exo at a ratio of 1:3 and left to incubate at room temperature for 18 h. Following Haney et al.'s method [19], ultrasonic waves (UWs) were employed to generate transient pores in the PDL-Exo membranes. The Emo@PDL-Exo mixture was placed in an ice bath and then exposed to UWs with a power of 2 kHz for 6 cycles (4 s of pulse and 2 s of pause). The formation of Emo@PDL-Exo was confirmed using TEM.

### Bacterial strains and growth conditions

*S. mutans* ATCC 35668 and *L. acidophilus* ATCC 314 (purchased from the National Center for Genetic and Biological Resources of Iran) were cultured on mitis salivarius agar and MM10 sucrose agar, respectively for 24 h at 37 °C under 5% CO_2_ atmosphere. To prepare microbial suspensions, the bacteria were cultured in Brain Heart Infusion (BHI) broth (Merck, Germany) and incubated at 37 °C in presence of 5% CO_2_. The optical density of each bacterium during the logarithmic growth phase was then measured by spectrophotometer at a wavelength of 600 nm, and suspensions equivalent to 0.5 McFarland Standard (1.5 × 10^8^ CFU/mL) were prepared and the desired concentration was confirmed by culture.

### Determination of minimum inhibitory concentration (MIC) and minimum bactericidal concentration (MBC)

The Clinical and Laboratory Standards Institute (CLSI) M60 2020 guidelines [27] is used to determine the MICs and MBCs for Emo, PDL-Exo, and Emo@PDL-Exo. According to the CLSI guidelines, MIC refers to the minimum concentration of an antimicrobial substance that prevents visible growth of microorganisms. To determine the MIC, 100 µL of BHI broth was added to the wells of a 96-well microplate. Then, 100 µL of Emo, PDL-Exo, and Emo@PDL-Exo with a final concentration of 1000 µg/mL was separately added to the first wells of the first column. To dilute Emo, PDL-Exo, and Emo@PDL-Exo 1:2 compared to the previous well, 100 µL of the contents of the first well was added to the second well. This process is repeated up to the 10th well, and the contents of the last well were discarded. After that, 100 µL of microbial suspensions with a concentration of 1.5 × 10^6^ CFU/mL was added separately to the wells of the microplate. The microplates were then placed in an incubator at 37 °C in the presence of 5% CO_2_. After 24 h, the MICs of Emo, PDL-Exo, and Emo@PDL-Exo were determined by visual observation of the turbidity of the medium according to the CLSI guideline. Measurement of optical density using a spectrometer at 600 nm was used to confirm the observed turbidity and the MIC values.

MBC values were determined by spread plating 5 µL of the contents from the wells that showed no turbidity in the MIC test onto nutrient agar plates followed by incubation at 37 °C for 24 h under 5% CO_2_ atmosphere. The MBC was identified as the highest dilution (lowest concentration) at which no growth was observed on the plates.

### Light source

The experimental setup consisted of a blue laser (Laser Diode, ASHA, Iran) was used with a wavelength of 405 ± 10 nm and an output intensity of 150 mW/cm^2^ for a duration of 60 s. A 96-well microtiter plate was positioned vertically at a distance of 1 cm from the laser probe.

### Determination of endogenous ROS generation

In this study, 2′,7′-dichlorofluorescein diacetate (DCFH-DA) was used to quantify the production of endogenous ROS within the treated *S. mutans* and *L. acidophilus* cells [28]. The bacterial cells were obtained by centrifugation of a cell culture at 10,000 rpm for 15 min and washed with PBS. The cells were then resuspended in PBS to achieve a density of 10^8^ CFU/mL. The cell suspensions were treated with the MIC dose of Emo@PDL-Exo and exposed to blue laser for a duration of 60 s, while the control group (only bacterial cells) was left untreated. Following treatment, the cell suspensions were incubated with 10 μM DCFH-DA for 30 min at 37 °C in the dark. The fluorescence intensity was then measured using a fluorescence flow cytometry. An increase in fluorescence at 530 nm when the sample was excited at 485 nm indicated an increase in the redox state (i.e., accumulation of DCF in cells). The data were analyzed using FlowJo software (V10, Becton, Dickinson and Company, USA).

### Determination of the antimicrobial photoactivity of Emo@PDL-Exo

The growth rate of *S. mutans* and *L. acidophilus* was assessed using a previously described method [29]. Briefly, 100 μL of *S. mutans* and *L. acidophilus* suspensions having a density of 1.5 × 10^6^ CFU/mL was separately added to the wells of a 96-well microtiter plate. The bacterial cells were then exposed to the different experimental conditions as follows:

A. **Emo:** 100 μL of Emo at a concentration of 1/2 × MIC was added to the bacterial cells, and the microtiter plate was kept in the dark for 5 min.
B. **PDL-Exo:** 100 μL of PDL-Exo at a concentration of 1/2 × MIC was added to the bacterial cells, and the microtiter plate was kept in the dark for 5 min.
C. **Emo@PDL-Exo:** 100 μL of Emo@PDL-Exo at a concentration of 1/2 × MIC was added to the bacterial cells, and the microtiter plate was kept in the dark for 5 min.
D. **Blue laser:** 100 μL of BHI broth was added to the bacterial cells, and the bacterial cells were exposed to the blue laser with a wavelength of 405 ± 10 nm for 60 s.
E. **aPDT:** 100 μL of bacterial suspensions were separately treated by Emo, PDL-Exo, and Emo@PDL-Exo similar to groups A-C, respectively, and the bacterial cells were then exposed to the ultrasound waves similar to group E.
F. **Positive control:** 100 μL of 0.2% chlorhexidine (CHX) was added to the bacterial cells, and the microtiter plate was kept for 5 min.
G. **Negative control:** 100 μL of normal saline was added to the bacterial cells, and the microtiter plate was kept for 5 min.

Following every treatment, the bacterial cells underwent serial tenfold dilution using a medium, and the log_10_ CFU/mL values were computed by incubating the plates at 37 °C for 24 h in the presence of 5% CO_2_. To determine if there was bactericidal activity, the total count of CFU/mL in the original inoculum needed to be reduced by at least 99.9% (≥ 3 log_10_).

### Determination of minimum biofilm eradication concentration (MBEC) of aPDT using Emo@PDL-Exo

MBEC values of the different experimental conditions described in Sect. 2.8. for *S. mutans* and *L. acidophilus* were determined using a colorimetric method based on crystal violet (CV) assay as described in our previous study [30]. The mature *S. mutans* and *L. acidophilus* biofilms were prepared in wells of a 96-well microtiter plate, separately. For this purpose, a 200 μL suspension of *S. mutans* and *L. acidophilus* in BHI broth medium with a final concentration of 1.5 × 10^6^ CFU/mL was added to each well of a 96-well microtiter plate, separately. The plate was then incubated for 24 h at 37 °C in the presence of 5% CO_2_ with shaking at 150 rpm to allow bacterial biofilms to grow on the wells. After that, the medium was removed from each well, and unattached and planktonic bacterial cells were removed by washing with PBS (pH 7.4) three times. The bacterial cells in biofilms were then treated with different groups according to Sect. 2.8. After that, the microtiter plates were incubated for 24 h at 37 °C in the presence of 5% CO_2_, the media were removed from the wells, and the wells were gently washed with PBS to eliminate any unattached or loosely adherent bacterial cells. Then, each well was stained using CV dye (1%) for 20 min at 25 °C. The dye was removed from the wells and the wells were washed twice with PBS (pH 7.4). Ethanol (95%; 200 μL) was added into the wells and the microtiter plates were remained at room temperature for 15 min. Following discarded the content of wells, the microtiter plate was dried at 40 °C. Thereafter, acetic acid (33%; 200 μL) was added to each well and biofilm masses were quantified by measuring the optical densities (OD) value at 570 nm. To assess the treatment efficiency on biofilm killing/degradation, the percentage of biofilm killing/degradation was determined as follows:

$$\mathrm{Biofilm\ killing }({\text{degradation}})\mathrm{ \% }= (\mathrm{OD\ of\ untreated\ bacterial\ biofilms }-\mathrm{ OD\ of\ treated\ sample }/\mathrm{OD\ of\ untreated\ bacterial\ biofilms}) \times 100$$

### Determination of metabolic activity using XTT reduction assay

To assess the metabolic activity of *S. mutans* and *L. acidophilus* cells that had been treated with 1/2 × MIC doses of Emo, PDL-Exo, and Emo@PDL-Exo, followed by exposure to blue laser as mentioned above, the XTT (2,3-bis [2-methyloxy-4-nitro-5-sulfophenyl]-2H-tetrazolium-5-carboxanilide) reduction assay kit (Roche Applied Science in Indianapolis, IN, US) was utilized, as previously described by Coraça-Hubér et al. [31]. The treated bacterial suspensions were first centrifuged at 2,000 rpm for 10 min, and then the bacterial cell sediments were collected and dissolved in 200 μL of XTT-menadione-PBS solution. The samples were then incubated for 3 h at 37 °C. Next, the solutions were transferred to a new 96-well microtiter plate, and the absorbance was measured at 492 nm using a microplate reader.

### Bioinformatics analysis

#### Retrieval and molecular modeling of GtfB and SlpA proteins

The National Center for Biotechnology Information (NCBI) database (https://www.ncbi.nlm.nih.gov/.) was searched for the sequence retrieval of GtfB and SlpA proteins with accession numbers of WP_275248834 and WP_270747926, respectively. To find a suitable template, the amino acid sequences of Esp and Ace were examined using the Protein-Basic Local Alignment Search Tool (BLASTP) available at http://blast.ncbi.nlm.nih.gov/Blast. The crystal structures of the selected GtfB (PDB ID: 8FG8) and SlpA (PDB ID: 7QLE) were retrieved from RCSB Protein Data Bank (PDB).

#### Molecular dynamics simulation

The iMOD server (https://imods.iqfr.csic.es/) was used to predict the molecular dynamics simulation of GtfB and SlpA proteins. In evaluating the stability of the proteins, the iMOD server analyzed several factors, including the main-chain deformability plot, B-factor, eigenvalue, covariance map, and elastic network model.

#### Ligand preparation

The structure of Emodin (C_15_H_10_O_5_) in SDF format was obtained from the PubChem Compound Database (http://pubchem.ncbi.nlm.nih.gov).

#### Molecular docking

To conduct molecular docking analysis, any water molecules and heteroatoms, such as phosphate molecules were removed, and then docked GtfB and SlpA proteins with Emodin using the CB-Dock2 server, which is available at https://cadd.labshare.cn/cb-dock2. During the docking process, five conformers were taken into consideration for the ligands. The best docking complex between the target receptors and ligand was determined based on the conformations that had the most favorable free binding energy, which was indicated by the lowest value in kcal/mol.

### Determination of expression changes of virulence factors

Quantitative real-time polymerase chain reaction (qRT-PCR) was used to quantify the expression of genes involved in the biofilm formation of *S. mutans* (*gtfB*) and *L. acidophilus* (*slpA*). Immediately after treatment of *S. mutans* and *L. acidophilus* with sub-MIC doses of Emo, PDL-Exo, and Emo@PDL-Exo, followed by exposure to blue laser as mentioned above, the total RNAs were extracted using a Super RNA extraction kit (Anacell, Iran). The quality and quantity of RNAs were assessed by employing 1% agarose gel electrophoresis and a NanoDrop 8000 spectrophotometer (Thermo Fisher Scientific, Inc), respectively. To generate cDNA, a cDNA synthesis kit (Anacell, Iran) was used following the manufacturer's instructions.

The qRT-PCR was executed utilizing SYBR Premix Ex Taq II Kits (TaKaRa, Japan) and the ABI StepOne™ Real-Time PCR System. The procedure involved initial incubation at 95 °C for 5 min, followed by 40 cycles of 15 s at 95 °C and 58 s at 60 °C. The detection system software was employed to determine the threshold cycle values (CT), and the transcription levels of each gene were compared to the expression of the *16S rRNA* gene that was used as a reference gene. The data were analyzed based on the Ct numbers determined by the detection system software using the 2^−△△Ct^ method [32]. The qRT-PCR primers are listed in Table 1.

**Table 1:** Primer sequences used in this study

Genes		Sequences (5́ − 3́)^a^	Amplicon Size (bp)
***gtfB***	F	TGTTGTTACTGCTAATGAAGAA	103
	R	GCTACTGATTGTCGTTACTG	
***slpA***	F	ACTGCTAACAACACTCCAGC	153
	R	TTCAGCAGGTTTAACGGCAG	
***16S rRNA***	F	CCTACGGGAGGCAGCAGTAG	121
	R	CAACAGAGCTTTACGATCCGAAA	

*Abbreviations*: *F* Forward primer, *R* Reverse primer, and *bp* Base pair

^a^Nucleotides

### Statistical analysis

The experimental procedures were carried out, and the resulting data was presented as the mean ± SD. To determine statistically significant differences, a one-way ANOVA with Tuckey's multiple comparisons test was conducted using SPSS 25.0 (SPSS Inc., Chicago, IL, United States). A *P* value of less than 0.05 was considered significant, and the respective graphs display the individual values.

## Results

### Validation of PDL-Exo and Emo@PDL-Exo

**Fig. 1:**
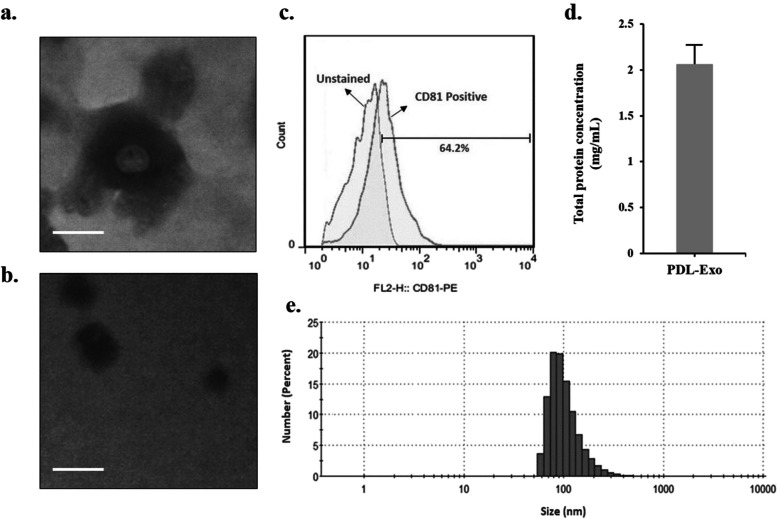
Characterization of PDL-Exo and Emo@PDL-Exo: **a** TEM micrograph of PDL-Exo (Scale bar = 100 nm), **b** TEM micrograph of Emo@PDL-Exo (Scale bar = 50 nm), **c** Flow cytometry analysis of Exo-specific marker CD81, **d** Total protein concentration of PDL-Exo, **e** Average particle size distribution of Emo@PDL-Exo

TEM results (Fig. 1a) confirmed that PDL-Exo maintains its intact, sphere-shaped vesicle morphology with an average diameter of < 100 nm. TEM also revealed an average diameter of approximately 110 nm with a spherical structure for Emo@PDL-Exo (Fig. 1b). CD81, an exosome-specific marker, was detected in the PDL-Exo sample (Fig. 1c). The protein concentration of PDL-Exo was determined using a standard protein concentration curve generated by sequential dilution of BSA with a specified concentration and the Bradford solution. The calculated concentration of PDL-Exo protein was 2060 μg/mL (Fig. 1d). Moreover, DLS analysis (Fig. 1e) showed a size distribution between 55 and 128 nm in diameter, with the majority of particles falling within the exosome size range (under 100 nm) when adjusted with TEM results.

### MIC doses of PDL-Exo, Emo, and Emo@PDL-Exo against cariogenic bacteria

**Fig. 2:**
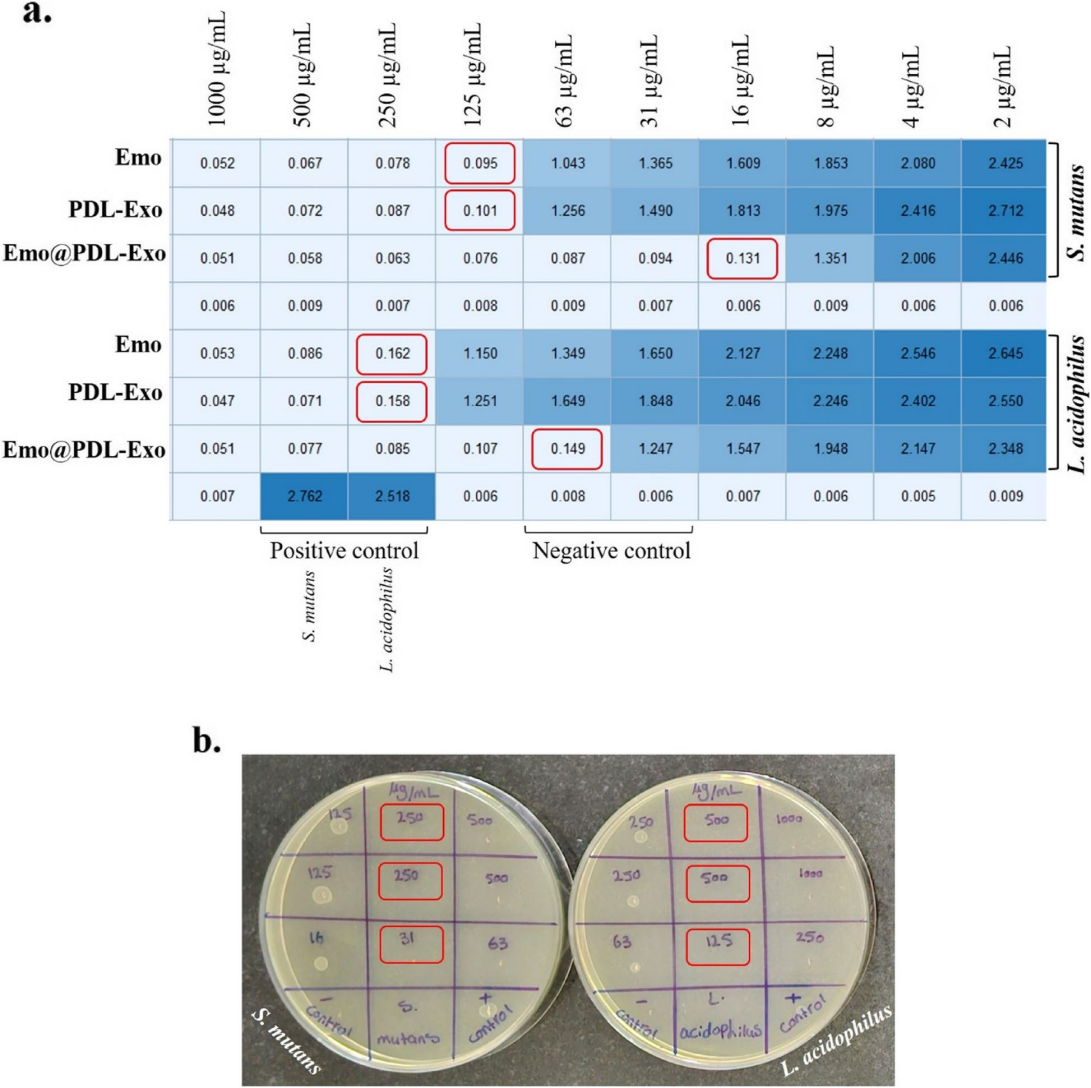
**a** Determination of minimum inhibitory concentration (MIC) after taking optical density using a spectrometer at 600 nm for the various dilutions of Emo, PDL-Exo, and Emo@PDL-Exo incubated with *S. mutans* and *L. acidophilus*; Red rectangle = MIC, **b** Determination of minimum bactericidal concentration (MBC) of Emo, PDL-Exo, and Emo@PDL-Exo against *S. mutans* and *L. acidophilus*; Red rectangle = MBC

Turbidity was observed after 24 h of incubation in the test wells containing Emo@PDL-Exo at concentrations ranging from 2 to 8 μg/mL, indicating the growth of *S. mutans*. On the other hand, there was no turbidity in concentrations of 16, 31, 63, 125, 250, 500, and 1000 μg/mL, demonstrating inhibition of bacterial growth. The wells containing Emo and PDL showed considerable turbidity at concentrations from 2 to 63 μg/mL, but no turbidity was observed at concentrations higher than 63 μg/mL. As a result, according to visual observation of the turbidity of the medium according to the CLSI guideline, the MIC values of Emo, PDL-Exo, and Emo@PDL-Exo for *S. mutans* were 125 μg/mL, 125 μg/mL, and 16 μg/mL, respectively (Fig. 2a). On the other hand, Emo-containing wells showed significant turbidity, at concentrations ranging from 2 to 125 μg/mL, indicating the growth of *L. acidophilus*. The well containing PDL-Exo exhibited considerable turbidity at concentrations less than 250 μg/mL. Turbidity was also observed in the wells containing Emo@PDL-Exo at concentrations from 2 to 31 μg/mL, indicating the growth of *L. acidophilus*, while no turbidity was seen in concentrations ranging from 63 to 1000 μg/mL. Therefore, according to visual observation of the turbidity of the medium according to the CLSI guideline the MICs for Emo, PDL-Exo, and Emo@PDL-Exo for *L. acidophilus* were 250 μg/mL, 250 μg/mL, and 63 μg/mL, respectively. Obtained results of optical density using a spectrometer at 600 nm were confirmed the observed turbidity and the MIC values of Emo, PDL-Exo, and Emo@PDL-Exo for *S. mutans* and *L. acidophilus* (Fig. 2a). The results revealed that the MBCs of Emo, PDL-Exo, and Emo@PDL-Exo for *S. mutans* were found to be effective at dilution of 250 μg/mL, 250 μg/mL, and 31 μg/ml, respectively (Fig. 2b). In the case of *L. acidophilus*, MBCs of Emo, PDL-Exo, and Emo@PDL-Exo were 500 μg/mL, 500 μg/mL, and 125 μg/ml, respectively (Fig. 2b).

### Measurement endogenous ROS generation using flow cytometry

**Fig. 3:**
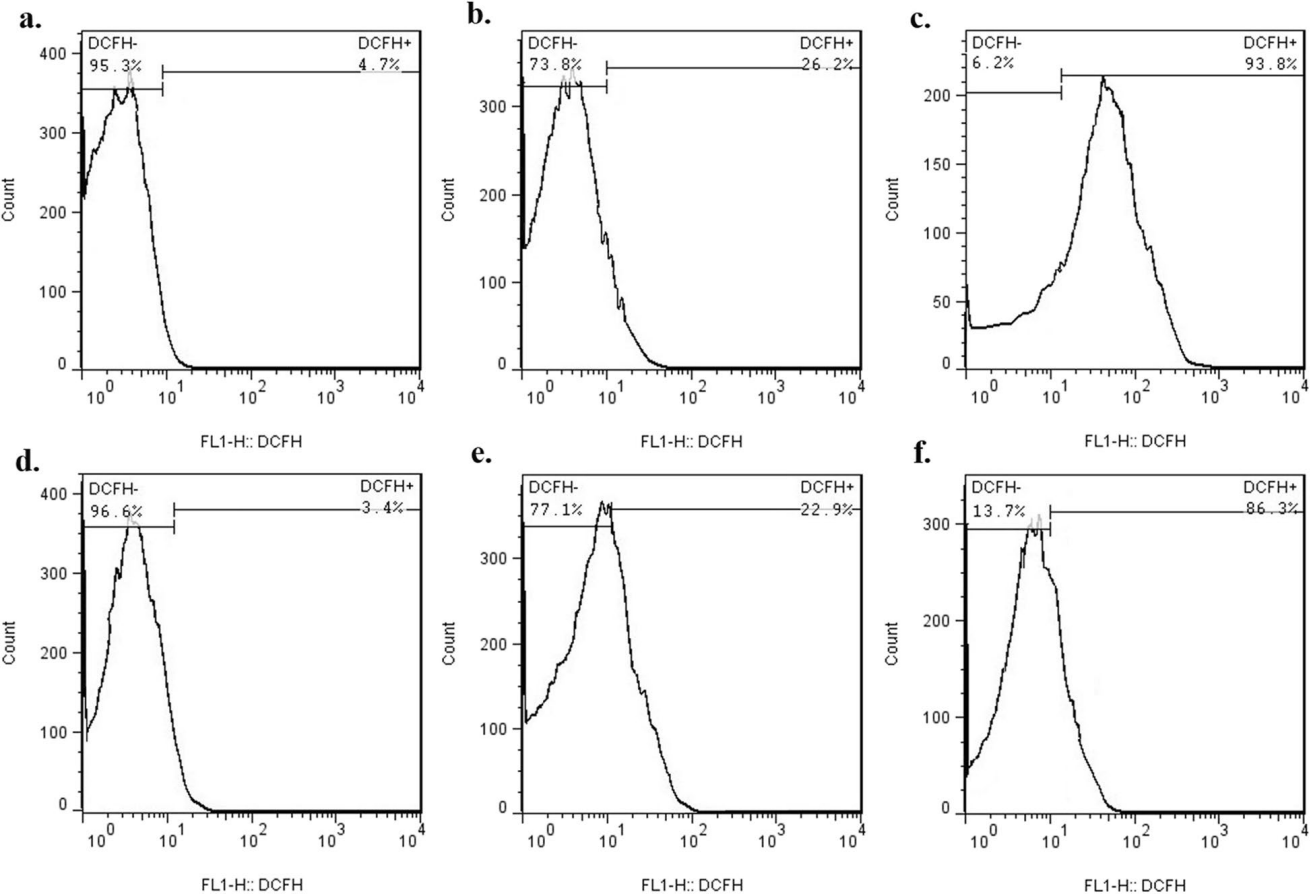
Histograms of endogenous reactive oxygen species (ROS) in: **a** *L. acidophilus* cells treated with normal saline (control group), **b** *L. acidophilus* cells treated with Emo@PDL-Exo, **c** *L. acidophilus* cells treated with aPDT using Emo@PDL-Exo, **d** *S. mutans* cells treated with normal saline (control group), **e** *S. mutans* cells treated with Emo@PDL-Exo, and **f** *S. mutans* cells treated with aPDT using Emo@PDL-Exo

Figure 3 depicts histograms of endogenous ROS generation in cariogenic bacteria that were subjected to different treatment groups. The results show that *L. acidophilus* treated with aPDT using Emo@PDL-Exo using aPDT exhibited a 20-fold higher proportion of cells with bright fluorescence and more than 13-fold higher intensity of total fluorescence compared to untreated *L. acidophilus* (Fig. 3c vs. a), as indicated by the level of DFC-DA mediated fluorescence. Similarly, *S. mutans* treated with aPDT using Emo@PDL-Exo showed more than a 25-fold higher proportion of cells with bright fluorescence and sevenfold higher intensity of total fluorescence compared to untreated *S. mutans* (Fig. 3f vs. d), as determined by the level of DFC-DA mediated fluorescence.

### Cell viability of cariogenic bacteria

**Fig. 4:**
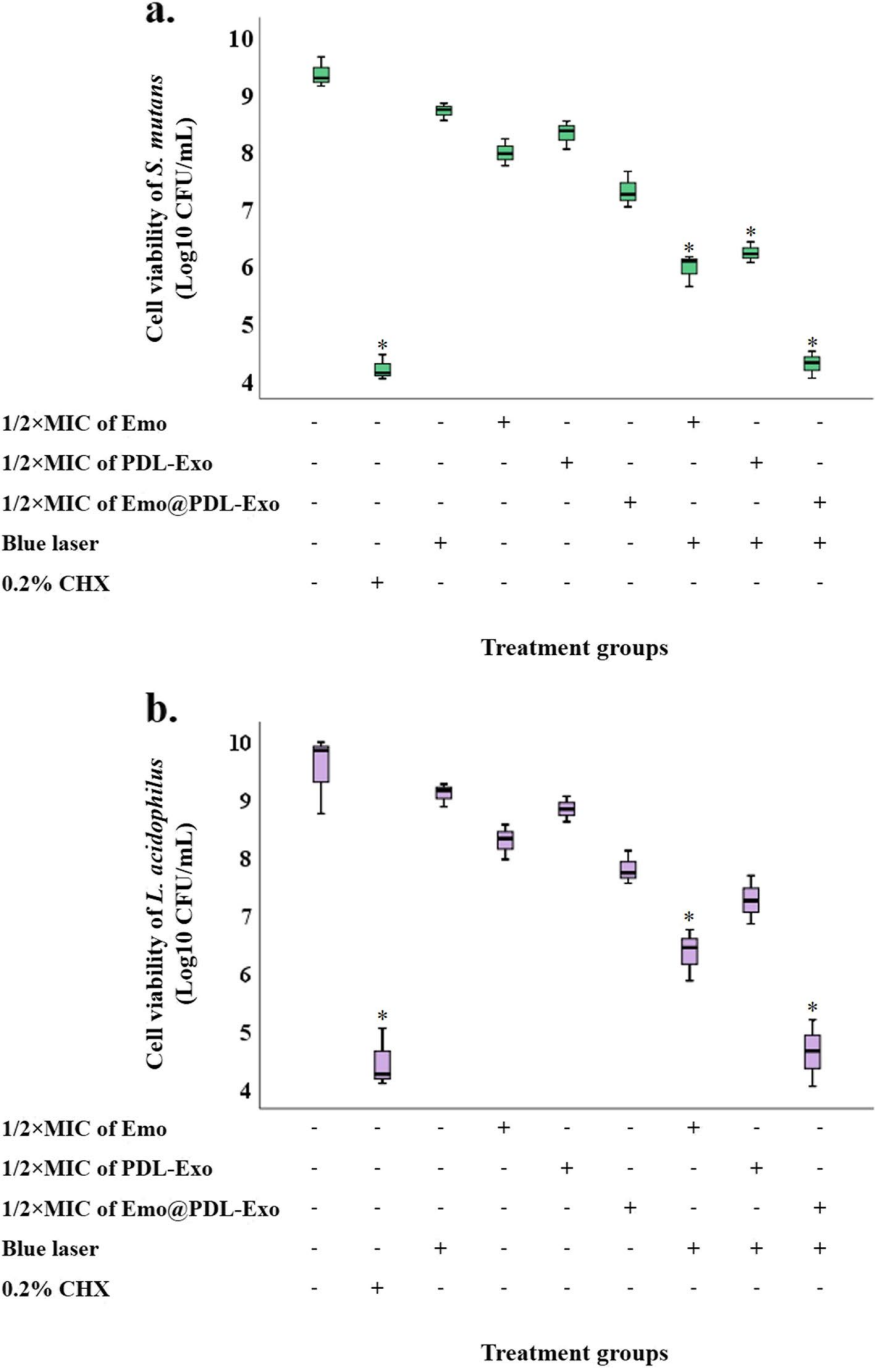
Effect of different treatment groups on cell viability of: **a*** S. mutans*; **b** *L. acidophilus*. *Significantly different from the control group (no treatment), *P* < 0.05

Figure 4 displays the results of Log_10_ CFU/mL of *L. acidophilus* and *S. mutans* after each treatment. The findings indicate that the cell viability of both cariogenic bacteria reduced following aPDT groups compared to the control group. The most effective bactericidal impact was observed at aPDT using 1/2 × MIC of Emo@PDL-Exo, resulting in a reduction in bacterial count of 4.90 and 5.07 log_10_ CFU/mL for *L. acidophilus* and *S. mutans*, respectively. There was no bactericidal activity (i.e., ≥ 3 log_10_ CFU/mL decrease) observed at 1/2 × MIC of Emo, PDL-Exo, and Emo@PDL-Exo alone for any strain. Furthermore, a non-significant decrease in log_10_ CFU/mL of both cariogenic bacteria was observed when treated with laser light alone.

### Anti-biofilm effects of different treatments on the cariogenic bacteria

**Fig. 5:**
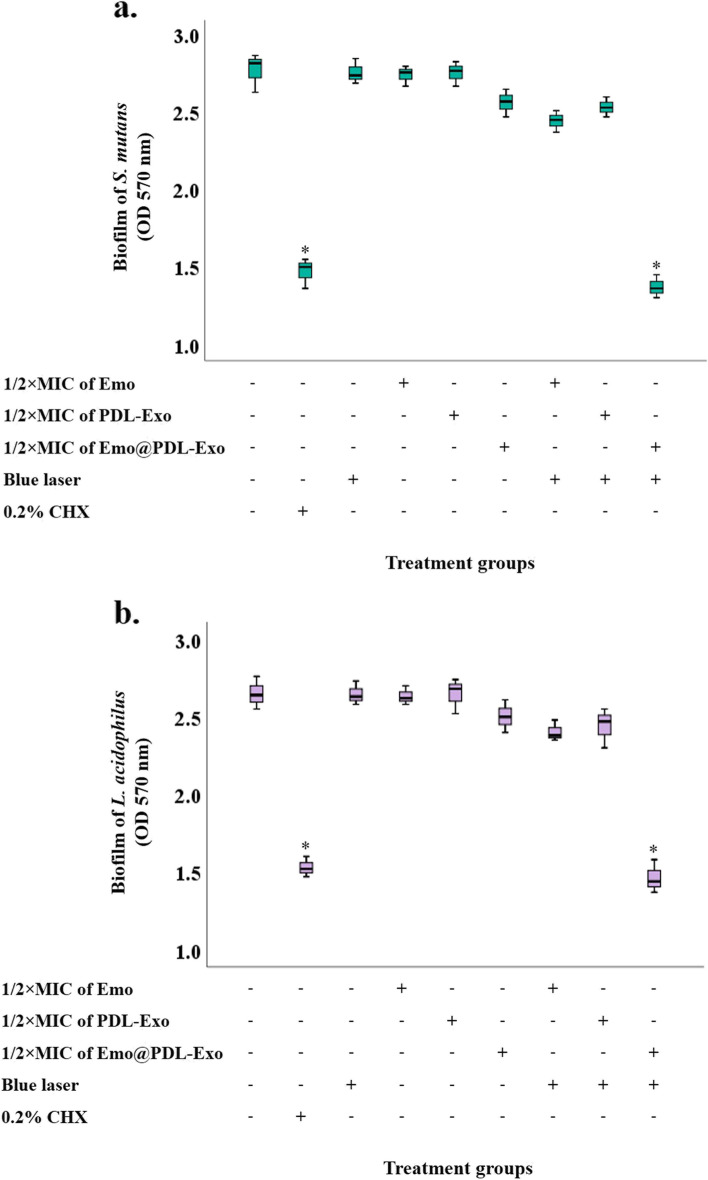
Effect of different treatment groups on biofilm of: **a*** S. mutans*; **b** *L. acidophilus*. *Significantly different from the control group (no treatment), *P* < 0.05

As shown in Fig. 5, the OD of untreated *L. acidophilus* and *S. mutans* biofilms as the control groups were 2.65 and 2.78, respectively. The biofilms reduction activity of aPDT using Emo@PDL-Exo against *L. acidophilus* and *S. mutans* biofilms (44.7% and 50.4%, respectively) were significantly higher than untreated biofilms as the control group (*P* < 0.05). There was no significant difference between biofilm reduction activity of aPDT using Emo@PDL-Exo against *L. acidophilus* versus *S. mutans* biofilms (*P* > 0.05; Fig. 5). As shown in Fig. 5, there were no significant differences in the reduction of *L. acidophilus* and *S. mutans* biofilms following Emo, PDL-Exo, Emo@PDL-Exo, blue laser, Emo plus blue laser, and PDL-Exo plus blue laser treatments compared to the untreated *L. acidophilus* and *S. mutans* biofilms as the control groups (all *P* > 0.05).

### Metabolic activity of treated cariogenic bacteria

**Fig. 6:**
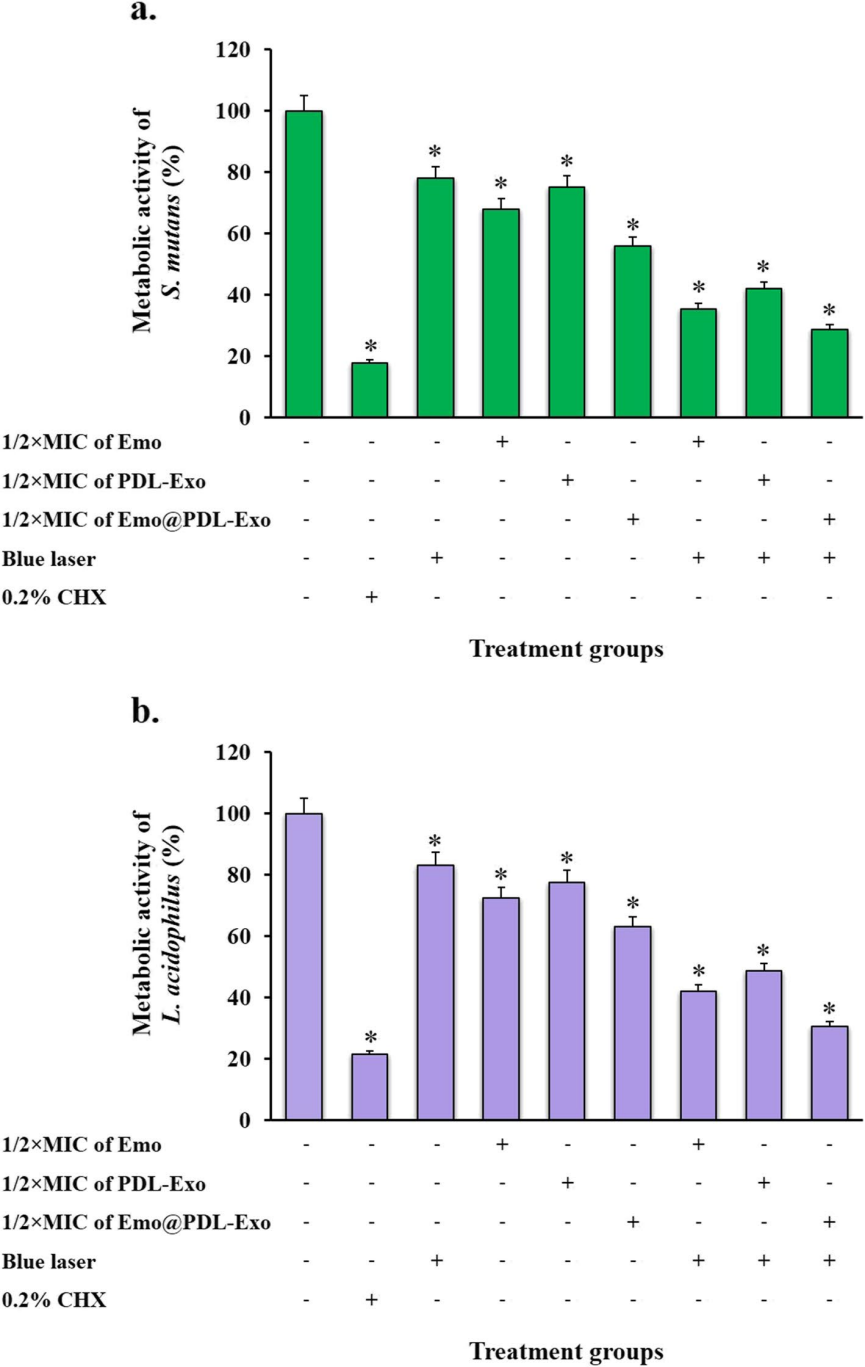
Effect of different treatment groups on metabolic activity of: **a*** S. mutans*; **b** *L. acidophilus*. *Significantly different from the control group (no treatment), *P* < 0.05

To measure metabolic activity in treated *S. mutans* and *L. acidophilus*, we employed the XTT reduction assay. According to the results, all aPDT treatment groups significantly reduced the metabolic activity of both bacteria (*P* < 0.05; Fig. 6). Figure 4 demonstrates that aPDT using sub-MIC of Emo@PDL-Exo was more effective in reducing the metabolic activity of *S. mutans* and *L. acidophilus* than the other treatment groups (*P* < 0.05). In this treatment group, the metabolic activity of *L. acidophilus* and *S. mutans* decreased by 58.3% and 71.2%, respectively (*P* < 0.05). Blue laser irradiation also displayed anti-metabolic activity on both cariogenic bacteria compared to the control group, but it was less than that observed in the Emo, PDL-Exo, and Emo@PDL-Emo groups at sub-MIC (*P* > 0.05). Furthermore, the metabolic activity of *L. acidophilus* and *S. mutans* after treatment with 0.2% CHX significantly decreased by 65.4% and 82.1%, respectively.

### Retrieval of GtfB and SlpA proteins

A similarity search was conducted against target proteins identified in *S. mutans* and *L. acidophilus* using BLASTP, revealing that GtfB and SlpA were structurally similar to proteins with PDB IDs 8FG8 and 7QLE, respectively. The protein alignment between GtfB and 8FG8 had a total score of 1753, with 58% and 98.37% query cover and identity, respectively. Similarly, the alignment between SlpA and 7QLE had a query cover, identity, and total score of 51%, 100%, and 315, respectively.

**Fig. 7:**
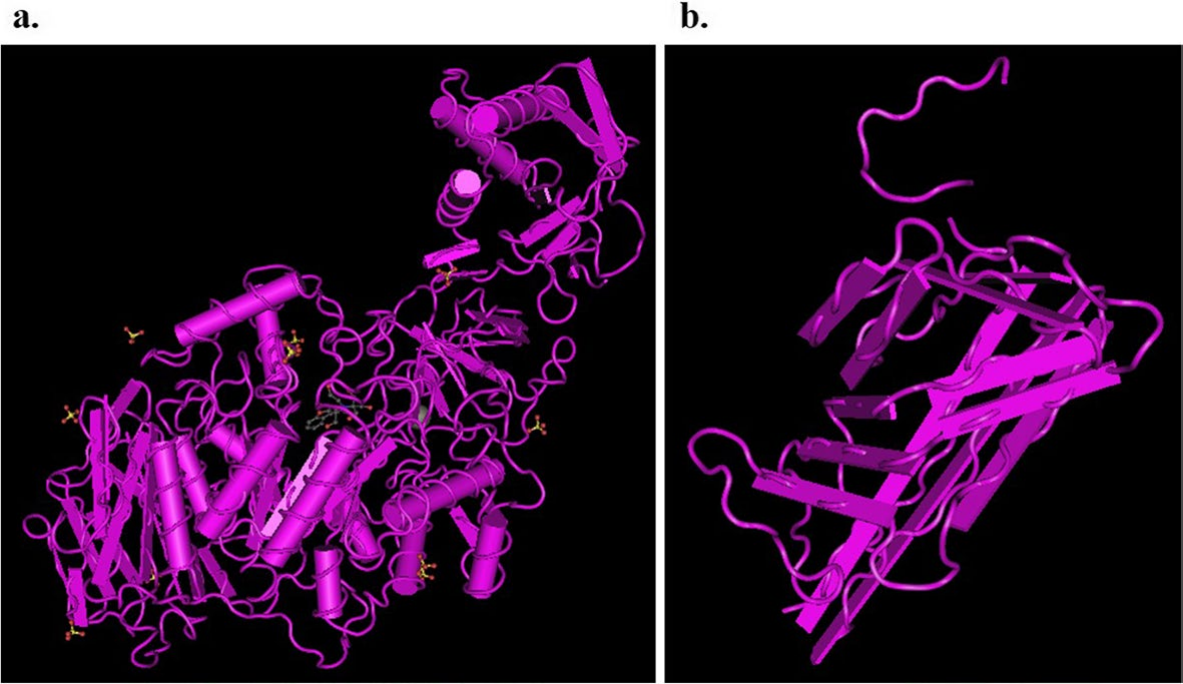
Three-dimensional structure of proteins: **a** GtfB (PDB ID: 8FG8), **b** SlpA (PDB ID: 7QLE)

The protein modeling for GtfB and SlpA was performed using the SWISS-MODEL web server. The molecular components of 8FG8, with a resolution of 2.35 Å, consist of one calcium ion (Ca^+2^), one (2Z)-2-[(2,4,5-trihydroxyphenyl)methylidene]-1-benzofuran-3(2H)-one (C_15_H_10_O_5_), one 2-[BIS-(2-hydroxy-ethyl)-amino]-2-hydroxymethyl-propane-1,3-diol (C_8_H_19_NO_5_), and ten Sulfate ion (O_4_S) (Fig. 7a). On the other hand, the molecular structure of 7QLE, with a resolution of 2.60 Å, is monomeric (Fig. 7b).

### Molecular dynamics simulations

**Fig. 8:**
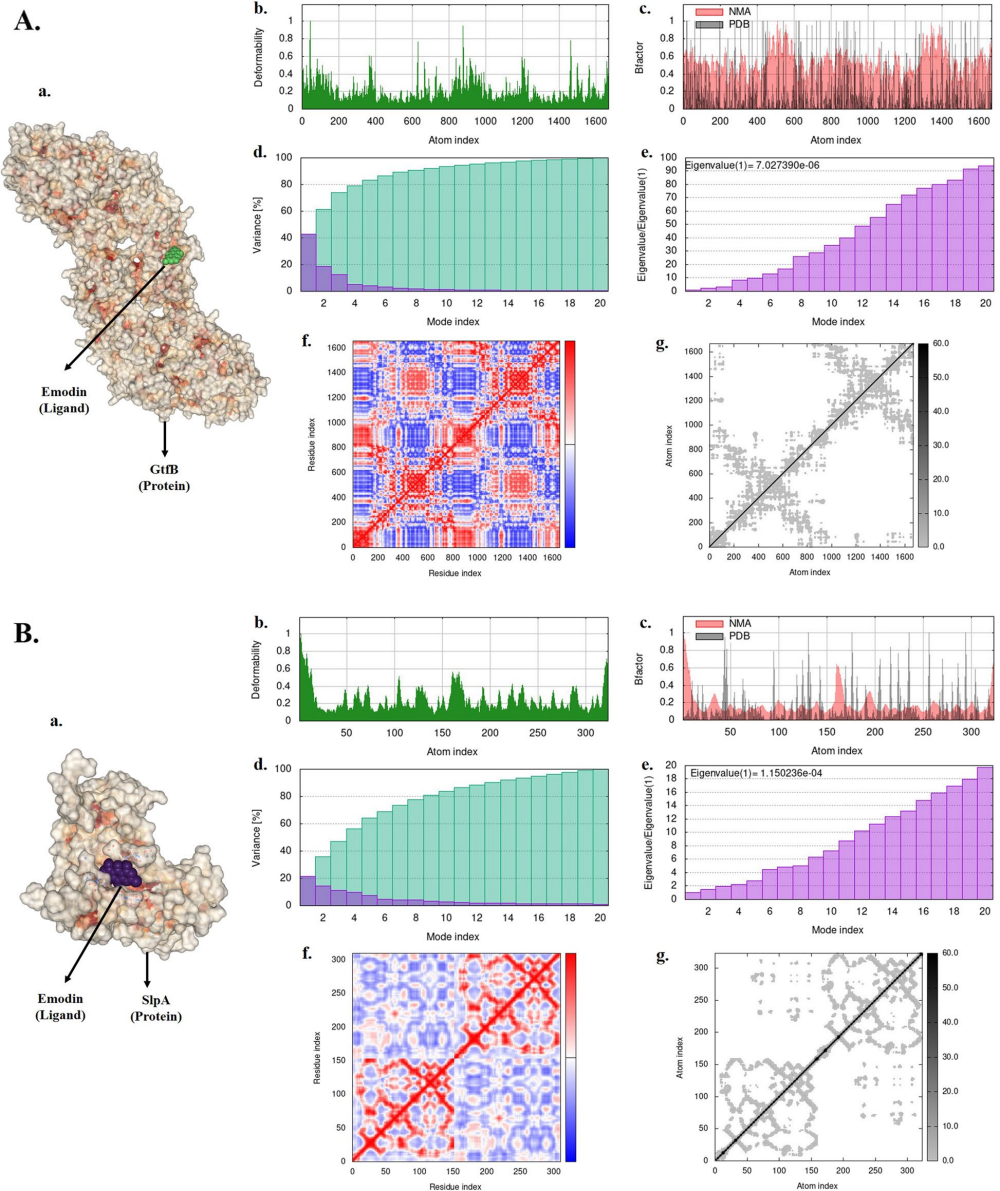
Molecular dynamic simulation: A GtfB, B. SlpA. a Protein–ligand complex, b Deformability, c B-factor values, d Variance (violet: individual variances, green: cumulative variances), e Eigenvalues, f Co-variance map (residues with correlated motions in red, uncorrelated motions in white, and anti-correlated motions in blue), and g Elastic network (darker grays indicate stiffer springs) of the complex

Figure 8 displays the results of our molecular dynamics simulation, which considered the docked complex of our ligand with receptor proteins. Images a of Fig. 8A and B display of our docked complex of proteins and ligand, while images b of show the deformability graph. Peaks in the deformity graph indicate high hinges and high deformability. Images c depict the main-chain deformability or B-Factor, which is a measure of the ability of a given molecule to deform at each of its residues. The variance plots are represented in images d, with individual variances shown in violet color and cumulative variance in green color. Images e display the eigenvalue of the complexes, which represents the motion stiffness of each normal mode. The value of the eigenvalue indicates the amount of energy required to deform the structure. A lower eigenvalue corresponds to easier deformation. Our docked complex exhibited eigenvalues of 1.150236e-04 and 7.027390e-06 for SlpA and GtfB, respectively. Images f show the covariance map, which displays the correlation motion between a pair of residues in red color, uncorrelated motion in white color, and anti-correlated motion in blue color. Finally, the elastic map of the docked complexes is also shown in images g, with each dot in the graph representing one spring within the respective atoms pair. The dots are colored based on stiffness, with dark grey dots indicating stiffer springs and vice versa. Based on the results of the molecular dynamic simulation, it can be inferred that the proposed proteins are stable.

### Molecular docking

**Fig. 9:**
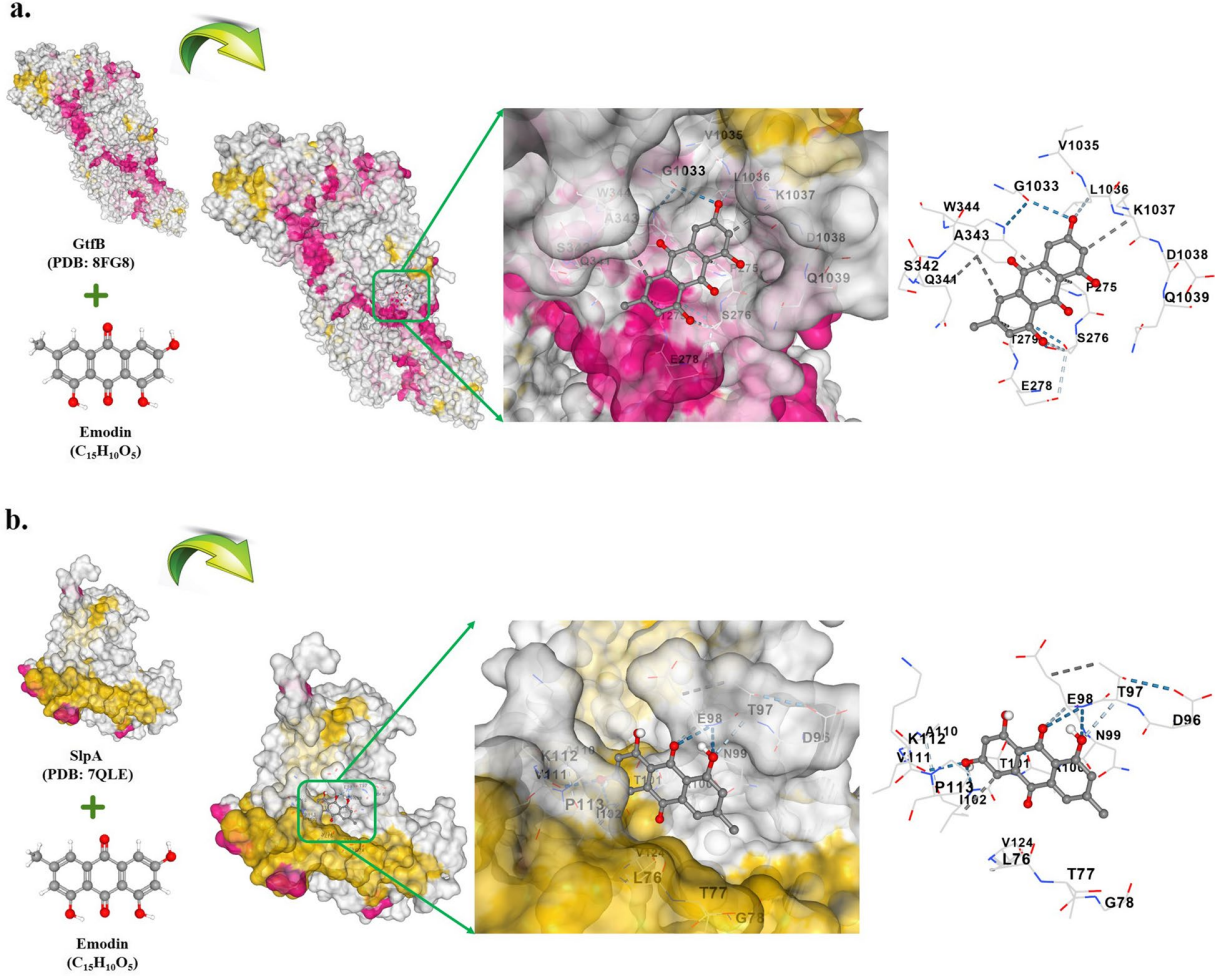
Depiction of docked ligand–protein complex along with interaction of the amino acid residues of the protein with ligand: **a** GtfB-Emodin, **b** SlpA-Emodin

The molecular docking study revealed that Emodin had a strong binding affinity towards the proposed proteins. The binding energies of Emodin to GtfB and SlpA were -8.2 and -7.4 kcal/mol, respectively (Fig. 9a and b). The residual amino acid interactions of GtfB with the ligand included LYS339, THR340, ASN345, SER346, ASP347, LEU1028, GLY1029, ARG1030, ASN288, ALA289, GLY292, ILE293, ASN294, and THR296. Meanwhile, the hydrogen interaction between the ligand and amino acid residues of SlpA involved THR101, THR103, ALA104, ALA105, GLY106, THR123, VAL124, THR125, THR103, ALA104, ALA105, GLY106, SER107, THR108, and ASN165. Therefore, it can be concluded that the GtfB-Emodin and SlpA-Emodin complexes are stable with high binding affinity.

### Effects of different treatment groups on gene expression profiles

**Fig. 10:**
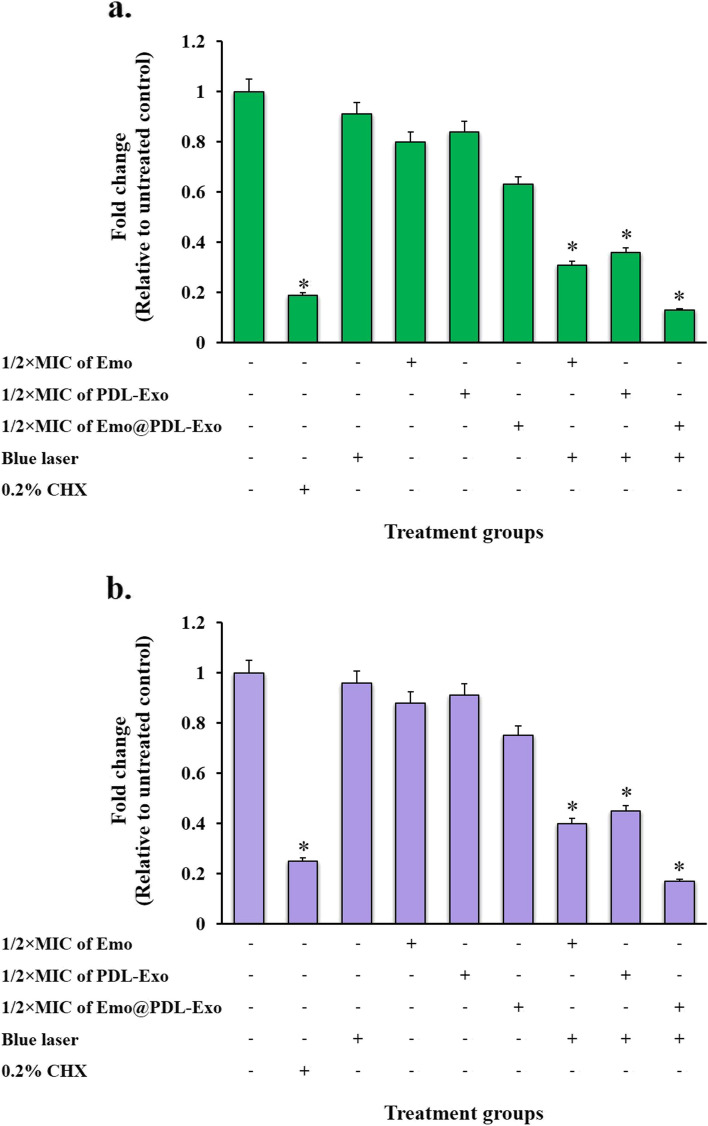
Effect of natural photosensitizers-mediated aPDT on gene expression of: **a** *gtfb*, **b** *slpA*. *Significantly different from the control group (no treatment), *P* < 0.05

To investigate the impact of aPDT using Emo@PDL-Exo on the virulence factors associated with the formation of biofilms by *S. mutans* and *L. acidophilus*, we conducted qRT-PCR to measure the abundance of *gtfB* and *slpA* mRNA expression. The mRNA levels of each gene were normalized to an internal control, and then the expression levels in treated cells were compared to those in untreated cells, which were set as the reference value of 1. Our results in Fig. 10 showed significant differences in the levels of *gtfB* and *slpA* mRNA transcripts among the various treatment groups. Notably, the sub-MIC doses of Emo@PDL-Exo plus blue laser resulted in a significant downregulation of *gtfB* and *slpA* mRNA expression by 5.6- and 4.2-folds, respectively, in both *S. mutans* and *L. acidophilus* (*P* < 0.05). However, the sub-MIC groups of Emo, PDL-Exo, and Emo@PDL-Exo alone did not exhibit any effect on the expression of *gtfB* and *slpA* genes (*P* > 0.05). Moreover, exposure to blue laser alone led to a downregulation of *gtfB* and *slpA* mRNA expression by 0.83- and 0.54-fold, respectively, but the difference was not statistically significant compared to the control group (*P* > 0.05).

## Discussion

Dental caries poses a serious global challenge and affect people of all ages in both developed and developing countries [33]. Biofilm formation by *S. mutans* and *L. acidophilus* plays a crucial role in the development of dental caries. The extracellular polymeric substances matrix provides protection for the bacteria against environmental stresses and antimicrobial agents, allowing them to colonize and persist in the oral cavity. As treatments for dental caries are expensive and eradication is often difficult, prevention is considered the most desirable and recommended strategy [33]. However, due to the growing resistance to synthetic antimicrobials, adjuvant therapeutic approaches are being sought. One of these methods is aPDT, whose antimicrobial and anti-biofilm effects have been proven in both in vitro and in vivo studies against various microbial pathogens [34–37]. One of the important factors in aPDT is the photosensitizer, which can be incorporated into biological nanocarriers to enhance the effectiveness of aPDT [38, 39].

In the current study, we used Emo@PDL-Exo as a photosensitizing agent. Emodin is a natural compound found in the roots and barks of various plants, which has shown promising antimicrobial activity against a range of microorganisms. However, its clinical application has been limited by its poor solubility and bioavailability. One approach to address this issue is to use exosome as a delivery system for Emodin. Exosomes have gained increasing attention in recent years as potential therapeutic agents due to their ability to deliver cargo to target cells [40]. One promising application for exosome is as a nano-biocarrier for drug delivery, including for antimicrobial therapies. Exosome-based delivery systems have been investigated as a way to increase the efficacy of antimicrobial agents, including those used in aPDT [41]. The biological impact of exosomes is influenced by the cellular source and the physiological state of the donor cells [42]. PDL stem cells, which are a type of mesenchymal stem cell found in the connective tissue that surrounds teeth, have gained attention in this regard. PDL stem cells are more broadly accessible, have fewer ethical concerns, and are less expensive in comparison to other stem cell types [42–44].

Currently, the investigation of using Exos as a delivery system for antimicrobial agents is still in its preliminary phases. Li et al. [45] investigated the antimicrobial potential of angiopep-2 modified exosomes load rifampicin (ANG-Exo-RIF) on *Mycobacterium tuberculosis* strain H37Rv. Their findings revealed that the MIC of RIF, Exo-RIF, and ANG-Exo-RIF was 0.25 μg/mL against the H37Rv strain, indicating that the encapsulation of rifampicin in exosomes did not affect its antibacterial properties. Notably, their study also demonstrated that free exosomes did not exhibit any antibacterial activity. Yang et al. [46] found that exosomes as carriers of antibacterial drugs can deliver drugs into cells and improve intracellular antibacterial activity. They evaluated the effectiveness of linezolid-Exo on Methicillin-Resistant *Staphylococcus aureus* (MRSA)-infected macrophages in a mouse model of MRSA. The study demonstrated that delivering antibiotics through Exo was more effective in treating intracellular MRSA infections compared to using free linezolid antibiotics. Moreover, Qian et al. [47]. reported that exosomes loaded with silver nanoparticles had multiple benefits in treating skin lesions caused by *Pseudomonas aeruginosa* infection in mice. These benefits included promoting the deposition of collagen, stimulating angiogenesis and nerve repair, and exhibiting potent antimicrobial activity that significantly inhibited bacterial growth.

In this study, MIC doses of Emo, PDL-Exo, and Emo@PDL-Exo was investigated to identify the optimal concentration of the drug for use in combination with blue laser. This can help to enhance the efficacy of the treatment and reduce the risk of adverse effects. According to the findings, the MIC doses of Emo, PDL-Exo, and Emo@PDL-Exo against *S. mutans* were 125 μg/mL, 125 μg/mL, and 15.6 μg/mL, respectively, whiles 250 μg/mL, 250 μg/mL, and 62.5 μg/mL were the MIC doses of Emo, PDL-Exo, and Emo@PDL-Exo against *L. acidophilus*.

To the best of our knowledge, no previous research has been done on the effect of aPDT using Emo@PDL-Exo on cariogenic bacteria. The findings of the present study showed that aPDT using 1/2 × MIC of Emo@PDL-Exo result in a reduction in bacterial count of *L. acidophilus* and *S. mutans* by 4.90 and 5.07 log_10_ CFU/mL, respectively. Furthermore, the obtained data indicated the aPDT using 1/2 × MIC of Emo@PDL-Exo eradicated 44.7% and 50.4% of mature *L. acidophilus* and *S. mutans* biofilms, respectively. Also, the results of the current study showed that aPDT using 1/2 × MIC of Emo@PDL-Exo was effective in removing biofilm compared to 0.2% CHX, which is an FDA-approved broad-spectrum antimicrobial mouthwash. However, the statistical analysis showed that their difference is not statistically significant. The extracellular matrix of biofilm plays an important role in the resistance of bacteria in the form of biofilm to antimicrobial agents by preventing the entry of antimicrobial agents into the biofilm structure. The extracellular matrix of the biofilm is composed of many compounds such as proteins, polysaccharides, and extracellular DNA [48]. Several studies have shown the efficiency of aPDT in the reduction and elimination of bacterial pathogens was observed to be linked to oxidative stress induced by reactive oxygen, weakening of the biofilm matrix, loss of adhesion, and changes in components [49]. Moreover, the metabolic activity of *L. acidophilus* and *S. mutans* treated with 1/2 × MIC of Emo@PDL-Exo plus blue laser was decrease decreased by 58.3% and 71.2%, respectively.

Herein, the generation of endogenous ROS was also assessed. The results showed that the production of endogenous ROS was noticeably higher in *S. mutans* and *L. acidophilus* treated with Emo@PDL-Exo alone and in combination with blue laser, as compared to the control group. Moreover, the amount of ROS generated in both cariogenic bacteria treated with aPDT was about four times greater than that produced in bacteria treated with Emo@PDL-Exo alone. ROS produced by exosomes can have both positive and negative effects, depending on the circumstances in which they are generated and the particular ROS species involved. While the production of ROS by exosomes can be beneficial in some physiological processes, such as in the regulation of signaling pathways and in defense against invading pathogens, it can also cause damage to cellular components like proteins, lipids, and DNA, and contribute to the development of diseases if produced in excess. Exosomes can also enhance the immune response by stimulating the production of ROS, which can help eliminate pathogens and reduce inflammation [50–52]. Therefore, it is crucial to regulate and balance the production of ROS by exosomes for maintaining normal physiological processes.

In this study, we utilized molecular docking as a powerful computational/in silico approach to explore the binding affinity of Emodin to surface proteins of *S. mutans* and *L. acidophilus*. The data showed that Emodin had the high binding affinity against GtfB and SlpA proteins. We also used normal mode analysis mobility to examine the large-scale B-factor and mobility, as well as the stability of the ligand–protein complex. The iMOD server was used to analyze the internal coordinates, which depended on the protein–ligand structural interactions [53]. The results of our molecular dynamics study indicated that our complex exhibited a considerable degree of deformability, as well as a moderately low eigenvalue, indicating that it could be easily deformed. The variance map demonstrated a higher level of cumulative variances than individual variances. Additionally, the elastic network map yielded satisfactory results. Thus, results of molecular dynamic simulation suggested that our proposed proteins are stable.

Finally, the effect of aPDT using Emo@PDL-Exo on the expression of genes involved in the biofilm formation of both cariogenic bacteria was also evaluated. The findings revealed that the administration of Emo@PDL-Exo at sub-MIC in combination with blue laser led to a marked reduction in *gtfB* and *slpA* mRNA expression. Specifically, the expression of *gtfB* and *slpA* mRNA was downregulated by 5.6- and 4.2-folds, respectively.

While acknowledging the limitations of the current study, our future studies will focus on the impact of the aPDT using Emo@PDL-Exo on normal oral microbiota, where the selective elimination of pathogens and maintaining normal microbiota plays a critical role in oral health. We believe that the approach used in this study can be a useful strategy for delivering other photosensitizing agents against microbial pathogens involved in dental caries. As such, it is crucial for researchers to continue exploring ways to enhance the isolation and loading efficiency of exosome, as well as developing targeting strategies and obtaining regulatory approval for clinical use. Ongoing research and development in this area could lead to the development of new and innovative exosome-based drug delivery strategies for combating microbial infections in the future. Therefore, further investigations for clinical applications are suggested. Our future studies also will focus on the impact of the therapy on normal oral microbiota, where the selective elimination of pathogens and maintaining normal microbiota plays a critical role in oral health.

## Conclusion

In summary, our study demonstrates that aPDT using Emo@PDL-Exo can effectively reduce the log_10_ CFU/mL, biofilm activity, and metabolic potency of *S. mutans* and *L. acidophilus*. Molecular docking analysis indicates that Emodin has a strong binding affinity with GtfB and SlpA proteins. Additionally, aPDT treatment significantly reduced the expression levels of *gtfB* and *slpA* mRNA in comparison to the untreated group due to the increased endogenous ROS generation. These findings suggest that Emo@PDL-Exo-mediated aPDT may be a promising antimicrobial approach for further investigation. Future in vitro and in vivo experiments are recommended to assess the potential application of this approach on different microbial pathogens associated with dental caries.

## Data Availability

The data of this study is available from the corresponding author on reasonable request.

## Abbreviations

aPDT: Antimicrobial photodynamic therapy
CHX: Chlorhexidine
Emo: Emodin
Gtf: Glucosyltransferase Gtf
PDL: Periodontal ligament
PDL-Exos: Periodontal ligament stem cell-derived exosomes
ROS: Reactive oxygen species
Slp: Surface layer proteins
